# Quantitative cross-species comparison of serum albumin binding of per- and polyfluoroalkyl substances from five structural classes

**DOI:** 10.1093/toxsci/kfae028

**Published:** 2024-03-22

**Authors:** Hannah M Starnes, Thomas W Jackson, Kylie D Rock, Scott M Belcher

**Affiliations:** Department of Biological Sciences, North Carolina State University, Raleigh, North Carolina 27607, USA; Department of Biological Sciences, North Carolina State University, Raleigh, North Carolina 27607, USA; Department of Biological Sciences, North Carolina State University, Raleigh, North Carolina 27607, USA; Department of Biological Sciences, North Carolina State University, Raleigh, North Carolina 27607, USA

**Keywords:** binding affinity, differential scanning fluorimetry, *in vitro*, PFAS, species extrapolation

## Abstract

Per- and polyfluoroalkyl substances (PFAS) are a class of over 8000 chemicals, many of which are persistent, bioaccumulative, and toxic to humans, livestock, and wildlife. Serum protein binding affinity is instrumental in understanding PFAS toxicity, yet experimental binding data is limited to only a few PFAS congeners. Previously, we demonstrated the usefulness of a high-throughput, *in vitro* differential scanning fluorimetry assay for determination of relative binding affinities of human serum albumin for 24 PFAS congeners from 6 chemical classes. In the current study, we used this assay to comparatively examine differences in human, bovine, porcine, and rat serum albumin binding of 8 structurally informative PFAS congeners from 5 chemical classes. With the exception of the fluorotelomer alcohol 1H, 1H, 2H, 2H-perfluorooctanol (6:2 FTOH), each PFAS congener bound by human serum albumin was also bound by bovine, porcine, and rat serum albumin. The critical role of the charged functional headgroup in albumin binding was supported by the inability of albumin of each species tested to bind 6:2 FTOH. Significant interspecies differences in serum albumin binding affinities were identified for each of the bound PFAS congeners. Relative to human albumin, perfluoroalkyl carboxylic and sulfonic acids were bound with greater affinity by porcine and rat serum albumin, and the perfluoroalkyl ether acid congener bound with lower affinity to porcine and bovine serum albumin. These comparative affinity data for PFAS binding by serum albumin from human, experimental model, and livestock species reduce critical interspecies uncertainty and improve accuracy of predictive bioaccumulation and toxicity assessments for PFAS.

Per- and polyfluoroalkyl substances (PFAS) are a class of synthetic chemicals, comprising thousands of structurally diverse compounds with at least one fully fluorinated methyl or methylene group (OECD, 2021). Due to their stability, persistence, and widespread use, PFAS are ubiquitously detected in biological and environmental matrices (Cousins *et al.*, 2020, 2022; Guillette *et al.*, 2020, 2022; Kirkwood *et al.*, 2022; Kotlarz *et al.*, 2020; Rock *et al.*, 2023). Some PFAS are toxic to multiple organ systems, including the hepatic, renal, immune, reproductive, and nervous systems (Carlson *et al.*, 2022; Crawford *et al.*, 2023; Radke *et al.*, 2022). Within the body many PFAS partition to, and accumulate in, protein-rich tissues, with highest concentrations found in the blood, liver, and kidneys of most exposed species (Bischel *et al.*, 2011; Hoff *et al.*, 2003). The majority of PFAS research has focused on perfluorooctanesulfonic acid (PFOS) and perfluorooctanoic acid (PFOA), whereas physiochemical properties of thousands of chemicals in this heterogeneous class remain uncharacterized (Radke *et al.*, 2022).

Serum albumin is the most abundant protein in vertebrate blood, constituting over 50% of human blood proteins (Merlot *et al.*, 2014; Peters, 1995; Raoufinia *et al.*, 2016). Serum albumin is the primary blood transport protein for many endogenous and exogenous ligands, including but not limited to long-chain fatty acids, bile acids, bilirubin, steroids, hormones, ions, and many pharmaceuticals (Merlot *et al.*, 2014; Peters, 1995). Additionally, several perfluoroalkyl acids (PFAAs), including PFOS, PFOA, perfluorohexanesulfonic acid (PFHxS), perflurononanoic acid (PFNA), and perfluorodecanoic acid (PFDA), are bound and transported by albumin in human blood (Forsthuber *et al.*, 2020). Experimental toxicokinetic studies in animals have demonstrated that PFOS, PFOA, and PFHxS are similarly bound by serum albumin of cynomolgus monkeys and rats, with binding affinities comparable to those of many endogenous ligands and pharmaceuticals (Kerstner-Wood *et al.*, 2003; U.S. EPA, 2016a,b; Varshney *et al.*, 2010).

In addition to influencing ligand transport and distribution, albumin binding affinity is an important determinant of biological half-life and bioavailability for many ligands (Kragh-Hansen, 1981; Lexa *et al.*, 2014; Merlot *et al.*, 2014; Peters, 1995; Sleep *et al.*, 2013; Zorzi *et al.*, 2019). Increased albumin binding affinity has a pivotal role in prolonging the half-life of many bioactive compounds (Sleep *et al.*, 2013). Along with increasing biological half-life and delaying elimination, plasma protein binding also decreases the relative free-fraction of PFAS available for uptake in target tissues, highlighting the potentially multifaceted role of albumin-PFAS interactions in determining pharmacokinetic properties of individual PFAS congeners (Ascenzi and Fasano, 2010; Han *et al.*, 2003; Kragh-Hansen, 1981; Merlot *et al.*, 2014; Peters, 1995; Sheng *et al.*, 2020).

Physiologically based pharmacokinetic (PBPK) modeling conducted by Cheng and Ng (2017) has suggested that albumin concentration and binding affinity are among the most important predictive parameters in determining overall PFOA blood concentration and tissue distribution in the rat. Additional PBPK modeling studies found that the plasma free fraction of PFOS and PFOA is most likely to travel between body compartments, and is therefore a key determinant of cellular uptake (Cheng and Ng, 2017; Chou and Lin, 2019, 2021; Deepika *et al.*, 2021; Fàbrega *et al.*, 2014). For example, a study conducted by Gao *et al.* (2019) found an inverse correlation between binding affinity of human serum albumin (HSA) for PFAS congeners and placental transfer efficiency.

Although advanced computational approaches have been developed to predict PFAS bioactivity and toxicity, there is significant uncertainty in the results of these predictive models due to the lack of foundational physiochemical data describing PFAS-protein interactions (Cheng and Ng, 2017, 2018, 2019). To improve quantitative binding predictions, physiologically-based toxicokinetic models, and ultimately risk assessments that inform exposure limits to protect public health, there is a critical need for experimental data describing albumin-PFAS interactions with structurally diverse sets of PFAS (Fenton *et al.*, 2021).

In addition to a lack of physiochemical data, there are also translational data gaps that limit ability to extrapolate PFAS toxicity data from animal models to humans. Major cross-species differences exist in PFAS elimination rates, with half-lives for some PFAS that are orders of magnitude apart in humans compared with model organisms and livestock (Fenton *et al.*, 2021; Pizzurro *et al.*, 2019). Although estimates vary across studies, reported half-lives for PFOS and PFOA tend to be on the order of hours to weeks in rodents, days to months in livestock, and years in humans (Death *et al.*, 2021; Lau, 2015; Numata *et al.*, 2014; Pizzurro *et al.*, 2019; Russell *et al.*, 2013). As albumin binding plays a key role in regulating PFAS bioaccumulation and distribution, differences in albumin-PFAS binding may influence differences in biological half-lives across species (Cheng *et al.*, 2021; D’eon *et al.*, 2010; Fenton *et al.*, 2021).

Differential scanning fluorimetry (DSF) is an *in vitro* method used to compare changes in thermal denaturation of ligand-free and ligand-bound proteins that accurately defines binding affinities for protein-PFAS interactions (Jackson *et al.*, 2021; Niesen *et al.*, 2007). Along with being fit-for-purpose, the DSF assay is scalable to high-throughput formats in readably available PCR instrumentation, requires small reagent volumes, uses small amounts of protein, and as a result is cost-effective. This combination of properties is highly advantageous compared with other methods for measuring protein-PFAS binding affinity, including isothermal titration calorimetry, surface plasmon resonance, or mass spectrometry (Jackson *et al.*, 2021; Liu *et al.*, 2019; Niesen *et al.*, 2007; Vivoli *et al.*, 2014).

We previously demonstrated the usefulness of a novel DSF assay for evaluating HSA binding of PFAS, reporting relative binding affinities for 24 PFAS from 6 chemical classes (Jackson *et al.*, 2021). Reported serum albumin binding affinities for PFAS vary widely across studies using different methods, however affinity values reported in Jackson *et al*. (2021) are within the range of those previously reported for albumin binding to PFAS, several fatty acids and drugs (Lee and McMenamy, 1980; Ràfols *et al.*, 2014; Spector, 1975; Takehara *et al.*, 2009). Using this assay, we identified differences in binding mediated by differences in PFAS functional groups and subtle changes in perfluoroalkyl chain length, demonstrating the capability and sensitivity of DSF for determining binding affinity differences among closely related PFAS congeners (Jackson *et al.*, 2021). Due to the combined benefits of assay sensitivity and scalability for high-throughput analyses, this DSF assay is capable of filling critical gaps in describing the physiochemical properties of PFAS (Jackson *et al.*, 2021).

To address the lack of comparative species specific PFAS-protein binding data, we utilized DSF to comparatively evaluate relative binding affinities of serum albumin from human, laboratory rat, and livestock (cow and pig) species for a subset of 8 structurally informative PFAS. The PFAS analyzed included the 4 congeners with completed toxicity assessments by the US Environmental Protection Agency (EPA): PFOA, PFOS, perfluorobutanesulfonic acid (PFBS), and hexafluoropropylene oxide-dimer acid (HFPO-DA) (U.S. EPA, 2016c, 2021a,b). To evaluate the generalizability of trends observed in HSA-PFAS binding based on carbon chain length, perfluorocarboxylic acid (PFCA) and perfluorosulfonic acid (PFSA) congeners with 4- and 8-carbon alkyl chains were included. Further, we leveraged 2 computational tools, the EPA Sequence Alignment to Predict Across Species Susceptibility (SeqAPASS) application and a molecular docking approach, to identify species-specific amino acid sequence differences in serum albumin, and to gain predictive insights into the mechanistic basis for observed interspecies differences in albumin-PFAS binding affinities (Cheng *et al.*, 2021; LaLone *et al.*, 2016).

## Materials and methods

###  

####  

##### Chemicals and reagents

**Figure 1.**
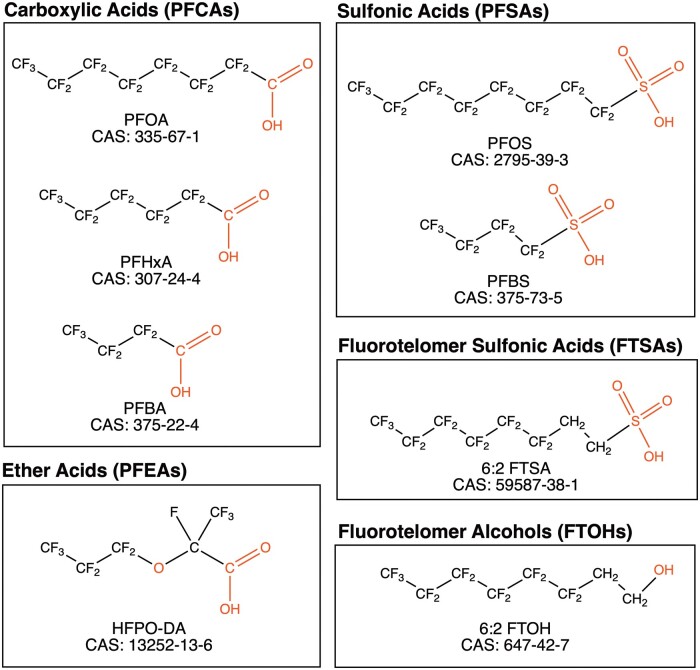
Chemical structures and Chemical Abstracts Service (CAS) registration numbers of PFAS congeners analyzed.

Aqueous solutions were prepared with sterile Milli-Q A10 water (18 Ω; 3 ppb total oxidizable organics). GloMelt (λEx = 468, λEm = 507 nm) and carboxyrhodamine (ROX; λEx = 588, λEm = 608 nm) dyes (CAT No. 33022-1) were purchased from Biotium (Fremont, California). Bovine serum albumin (BSA, purity ≥98%, fraction V, BP1605, Lot 053997), methanol (purity ≥99.9%, CAT No. A454-4), dimethyl sulfoxide (purity ≥99.9%, CAT No. 136-1, Lot 147002), Na_2_HPO_4_ (purity ≥99.2%, CAT No. S374-3, Lot 056560), NaCl (purity ≥100%, CAT No. S271-10, Lot 134874), and 4-(2-(2-hydroxyethyl)-1-piperazineethanesulfonic acid (HEPES, purity ≥99.%, CAT No. BP310-1, Lot 052975) were from Thermo Fisher Scientific (Waltham, Massachusetts). Rat serum albumin (RSA, purity ≥99%, A6414, Lot SLCF8334), and porcine serum albumin (PSA, purity ≥98%, A1830, Lot SLBZ2093) were from Sigma-Aldrich (St Louis, Missouri). Octanoic acid (OA, CAS 124-07-2, purity ≥95%) was from Alfa Aesar (Havermill, Massachusetts). Structures and chemical classes of the PFAS congeners analyzed are shown in Figure 1.

Perfluorobutanoic acid (PFBA, CAS 375-22-4, purity ≥99%), PFOA (CAS 335-67-1, purity ≥95%), and HFPO-DA/GenX (CAS 1352-13-6, purity ≥97%) were from Alfa Aesar (Havermill, Massachusetts). Perfluorohexanoic acid (PFHxA, CAS 307-24-2, purity ≥98%) and PFBS (CAS 375-73-5, purity ≥98%) were from TCI America (Portland, Oregon). PFOS (CAS 2795-39-3, purity ≥98%) was from Matrix Scientific (Columbia, South Carolina), and 1H, 1H, 2H, 2H-perfluorooctanol (6:2 FTOH, CAS 647-42-7, purity ≥97%) and 2H, 2H, 3H, 3H-perfluorooctane-1-sulfonate (6:2 FTSA, CAS 59587-39-2, purity ≥97%) were from Synquest Laboratories (Alachua, Florida).

##### PFAS and albumin preparation

PFAS stock solutions (10 or 20 mM depending on solubility) were prepared in aqueous HEPES-buffered saline (HBS; final concentrations were 140 mM NaCl, 25 mM HEPES, 0.38 mM Na_2_HPO_4_, pH 7.4) and appropriate solvent when necessary (Table 1). Calculated binding affinity values are not altered by the presence of dimethyl sulfoxide or methanol in the buffer of vehicle control and experimental samples (Jackson *et al.*, 2021). Serum albumin stocks (1 mM) were prepared by reconstituting lyophilized stocks in HBS, with expected protein concentrations confirmed using the colorimetric Bio-Rad DC Protein Assay (Hercules, California).

**Table 1.** Test chemical stocks

Chemical	Concentration (mM)	Solvent
PFBA	20	HBS
PFBS	20	HBS
PFHxA	20	HBS
HFPO-DA	10	HBS
6:2 FTSA	10	HBS
PFOA	10	HBS+50% DMSO
PFOS	10	HBS or HBS+50% DMSO
6:2 FTOH	10	HBS+30% DMSO
Octanoic acid	10	HBS+30% MeOH

Abbreviations: HBS, HEPES-buffered saline; DMSO, dimethylsulfoxide; MeOH, methanol.

##### DSF thermal shift assays

Individual assays were performed in sealed optical 96-well reaction plates (MicroAmp Fast, Applied Biosystems) at a final volume of 20 μL as previously described (Jackson *et al.*, 2021). A minimum of 2 independent plates were run for each protein-ligand combination, with 3 replicates per sample on each plate. Controls on each plate included a no protein control, matching vehicle control (no ligand), and 3 concentrations of OA (1, 2, 3 mM) and/or PFHxA (0.3, 1, 3 mM) as positive controls. The concentration of albumin that yielded maximal signal-to-noise ratio was optimized for each species. Final protein concentrations for each assay were 0.13 mM for HSA, 0.18 mM for BSA, 0.11 mM for PSA, and 0.18 mM for RSA. Changes in protein concentration in the assay did not result in detectable changes in protein melting temperature (*T*_m_). For concentration-response analyses, PFAS stock solutions were serially diluted into HBS.

##### Comparative amino acid sequence alignment

The SeqAPASS tool (https://seqapass.epa.gov/seqapass/; v. 6.1) was used to compare albumin primary amino acid sequences across species, with more targeted analysis comparing conservation of specific amino acid residues involved in ligand binding (LaLone *et al.*, 2016). For level 1 analysis, HSA (NCBI AAA98797.1) was used as the query protein to assess conservation of the complete amino acid sequence of BSA (NCBI P02769.4), PSA (NCBI ABM92961.1), and RSA (NCBI NP_599153.2). Level 3 analysis evaluated cross-species conservation of key amino acid residues in Sudlow sites I and II (Ascenzi and Fasano, 2010; Chen and Guo, 2009; Sudlow *et al.*, 1975, 1976). An amino acid substitution was identified in the Sudlow I site of BSA compared with HSA, and was further investigated using the DUET web-based tool (https://biosig.lab.uq.edu.au/duet/stability), which predicts the impacts of specified amino acid mutations on protein stability (Cheng *et al.*, 2021; Pires *et al.*, 2014). The crystal structure for HSA (PDB ID 1AO6) was submitted to DUET, and a mutation was specified to alter the amino acid residue at site 211 from phenylalanine (found in HSA) to leucine (found in BSA) in Sudlow site I of BSA.

##### Molecular docking

To further investigate interspecies differences in albumin-PFAS binding, Autodock Vina (v. 1.2.0) was used for molecular docking analyses (Eberhardt *et al.*, 2021; Trott and Olson, 2009). The RCSB Protein Data Bank (PDB; https://www.rcsb.org/) was used to obtain high resolution (≤2.5 Å) 3-dimensional protein structures. To assess HSA-PFAS docking, we used 3 monomeric structures previously chosen by Ng and Hungerbuhler (2015) to evaluate HSA-PFAA docking: HSA complexed with myristic acid (1E7G), HSA complexed with PFOS (4E99), and an intermediate conformation between the 2 (1H9Z) (Bhattacharya *et al.*, 2000; Luo *et al.*, 2012; Ng and Hungerbuehler, 2015; Petitpas *et al.*, 2001). Only one high-resolution BSA structure was available in PDB (4F5S), which was selected for use in our experiments (Bujacz, 2012). As 4F5S is a dimeric structure, one of the monomers was removed from the PDB file before docking. As of the date of publication, there were no crystal structures of PSA or RSA available in PDB. For comparative purposes, we followed the protocol for molecular docking described by Ng and Hungerbuhler (2015), with the exception that PFAS ligand files were downloaded as 3-dimensional SDF conformer files from NCBI PubChem database (https://pubchem.ncbi.nlm.nih.gov/), and converted to PDB format using PyMOL (Schrödinger L.L.C., 2017). Protein and ligand structures were prepared for docking using AutoDock Tools (v.1.5.7) as previously described (Morris *et al.*, 2009; Trott and Olson, 2009). Binding site boundary dimensions for 6 sites, defined by Ng and Hungerbuhler (2015), were selected using the Grid Box feature in Autodock Tools.

To evaluate experimental success of computational docking, we redocked PFOS to the crystallized HSA protein (PDB 4E99), and used PyMOL to measure the root mean square deviation (RMSD) between our docking prediction and the ligand position in the crystal structure (Cheng and Ng, 2018; Ng and Hungerbuehler, 2015; Schrödinger L.L.C., 2017). Calculated RMSD values below 2 Å confirmed that Autodock Vina was able to successfully predict binding conformations for HSA and PFOS (Li *et al.*, 2021; Ng and Hungerbuehler, 2015).

##### Data analysis and statistics

Raw DSF assay data, reported in relative fluorescent light units, were exported to Excel (Microsoft), and statistical analyses were performed using Prism (v.9.4.0, GraphPad Software Inc., San Diego, California), SPSS v28 (IBM, Armonk, New York), or R statistical environment (R Core Team, 2022). Melting temperature (*T*_M_) of albumin was defined as the temperature at which the maximum change in fluorescence occurs (Jackson *et al.*, 2021). PFAS concentration-response curves were smoothed (Savitzky and Golay, 1964), and dissociation constant (*K*_d_) values calculated using a single-site ligand binding model previously described (Jackson *et al.*, 2021; Vivoli *et al.*, 2014). For the biphasic shift in melting temperature observed for BSA binding of PFOS, the percent occupancy was used to calculate binding affinity (Vivoli *et al.*, 2014).

A Kruskal-Wallis test with Dunn’s multiple comparisons test was used to assess differences in the baseline melting temperatures of albumins. Generalized linear modeling (GLM) with a log-link Gaussian distribution was used for comparative evaluation of binding affinities, with PFAS and species as fixed factors, in the *lme4* R package (Bates *et al.*, 2015). McFadden’s pseudo-*R*^2^ was calculated to evaluate goodness of fit for the GLM (Veall and Zimmermann, 1996). Effect sizes for main effects of PFAS, species, and PFAS x species interaction on binding affinity were calculated using the *domin* function of the *domir* statistical package in R. A general dominance statistic (GD) that represents the proportion of variance associated with each main effect in the model was reported (Luchman, 2023). The *emmeans* R package was used to evaluate contrasts of interest, with Sidak’s multiple comparisons correction, to compare *K*_d_ differences between HSA and other albumins for each PFAS congener, and to evaluate the intraspecies effect of carbon chain length on *K*_d_ by comparing binding of PFBA and PFOA, and PFBS and PFOS (Lenth, 2023). For tests in which differences between binding constants were found, effect sizes for the *post hoc* analyses were determined by calculating Cohen’s d (Lakens, 2013). Effect sizes described by Cohen’s d are defined as small if *d*≤.2, medium if *d*≥.5, and large if *d*≥.8 (Lakens, 2013). For all statistical analyses, significant differences in means are defined as *p*<.05.

For each protein-PFAS combination, molecular docking Δ*G* predictions for the top 9 binding modes in each of the 6 analyzed binding sites were averaged. The geometric mean was taken across all binding sites for each protein, to represent the average Δ*G* of binding for that protein to each PFAS congener. To assess the strength of the linear relationship between calculated *K*_d_ values and molecular docking Δ*G* predictions for each protein-PFAS combination, Pearson’s correlation coefficients were computed using GraphPad Prism.

## Results

###  

#### Species comparison of serum albumin melting temperatures

In the absence of ligand, the observed average *T*_M_ ± SEM for HSA was 71.3 ± 0.22°C, BSA was 62.6 ± 0.04°C, PSA was 71.3 ± 0.09°C, and RSA was 66.8 ± 0.18°C. A Kruskal-Wallis test with Dunn’s multiple comparisons test (H = 184.8, *p*<.0001) revealed that baseline melting temperatures across each albumin protein were significantly different from one another, with the exception of HSA and PSA (*p*>.999).

#### DSF determination of ligand binding affinity

**Figure 2.**
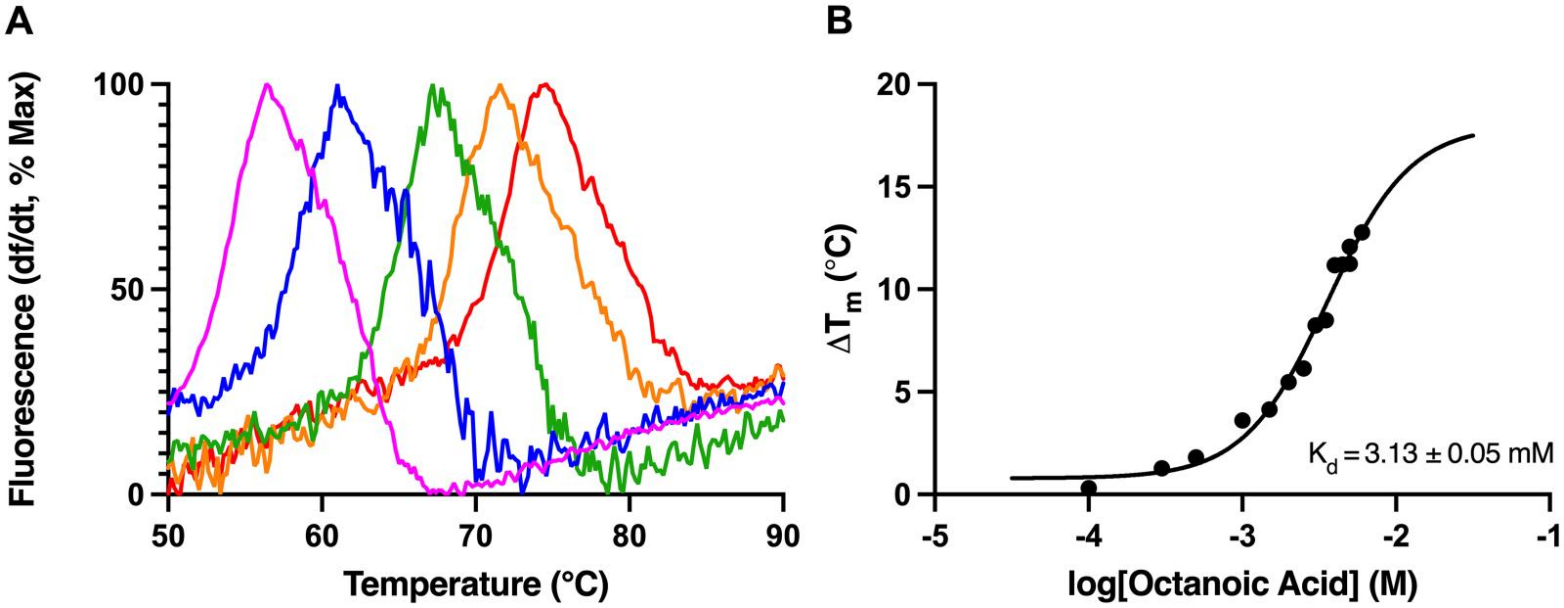
DSF analysis of BSA binding of octanoic acid. (A) Melt curves depicting changes in fluorescence for BSA with increasing concentrations of octanoic acid, normalized to percentage maximum fluorescence. Curves are plotted as the derivative of fluorescent signal divided by the derivative of time, as temperature is increased from 50°C to 90°C. Increasing concentrations of octanoic acid, from 0 (pink) to 5.5 mM (red) are depicted. (B) The change in melting temperature (Δ*T*_M_) of BSA as a function of the log-transformed concentration of octanoic acid from 0 to 6 mM. N = 6 replicates.

BSA binding affinity to OA was determined to confirm that DSF estimates for BSA binding were comparable to previously published values. Representative analysis of changes in BSA melting temperature induced by increasing concentrations of OA (0–5.5 mM) is shown in Figure 2A. The calculated *K*_d_ value (±SEM) of 3.13 ± 0.05 mM was in the expected range for fatty acid binding by bovine serum albumin (Figure 2B; Spector, 1975).

**Figure 3.**
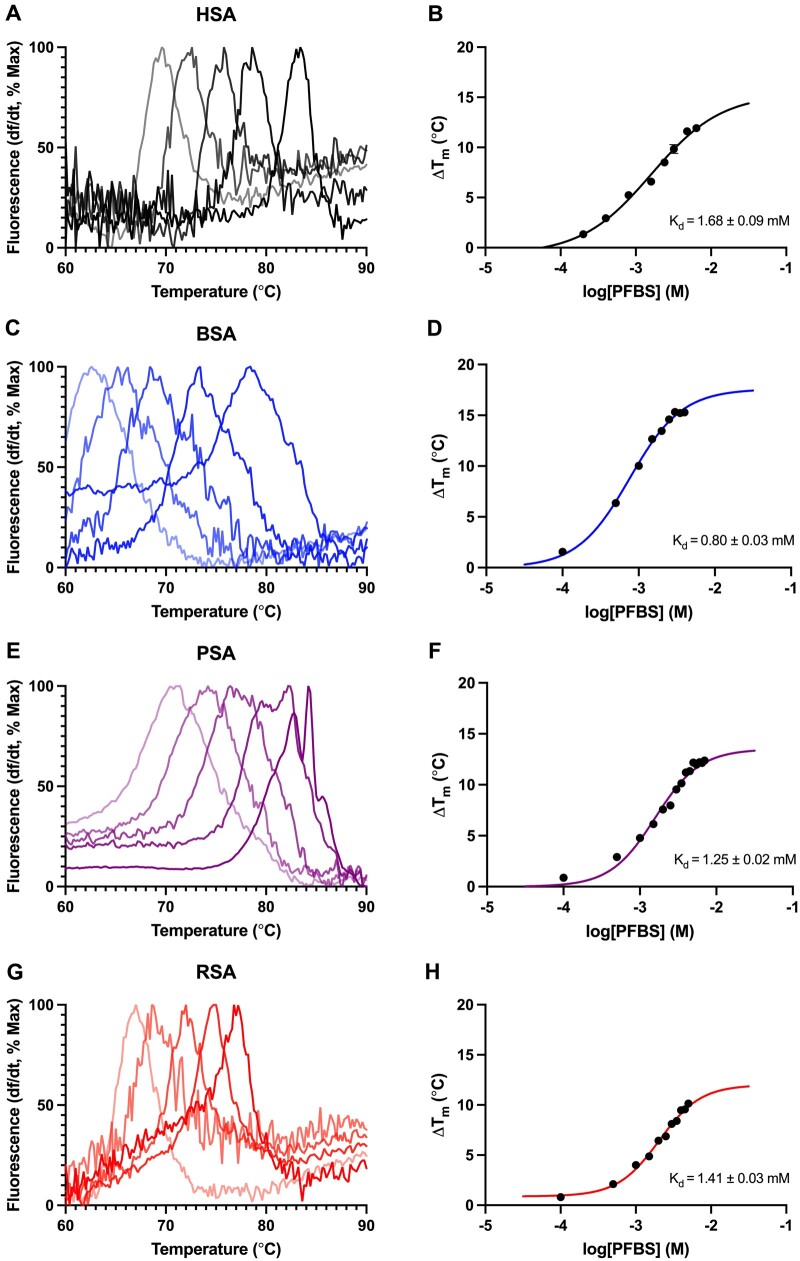
Melting temperature and concentration response analysis serum of albumin binding of PFBS. For each of the left-hand panels each curve depicts the change in fluorescent signal divided by change in time and is normalized to the percent maximum fluorescence for each albumin (A) HSA, (C) BSA, (E) PSA, and (G) RSA. Increased concentrations of PFBS are depicted by increased saturation of line color, as the temperature increased from 60°C to 90°C. The change in melting temperature (Δ*T*_M_) as a function of the log-transformed concentration of PFBS (M) and calculated binding affinity (*K*_d_) is shown in panels (B) HSA, (D) BSA, (F) PSA, and (H) RSA. N = 6 replicates.

**Figure 4.**
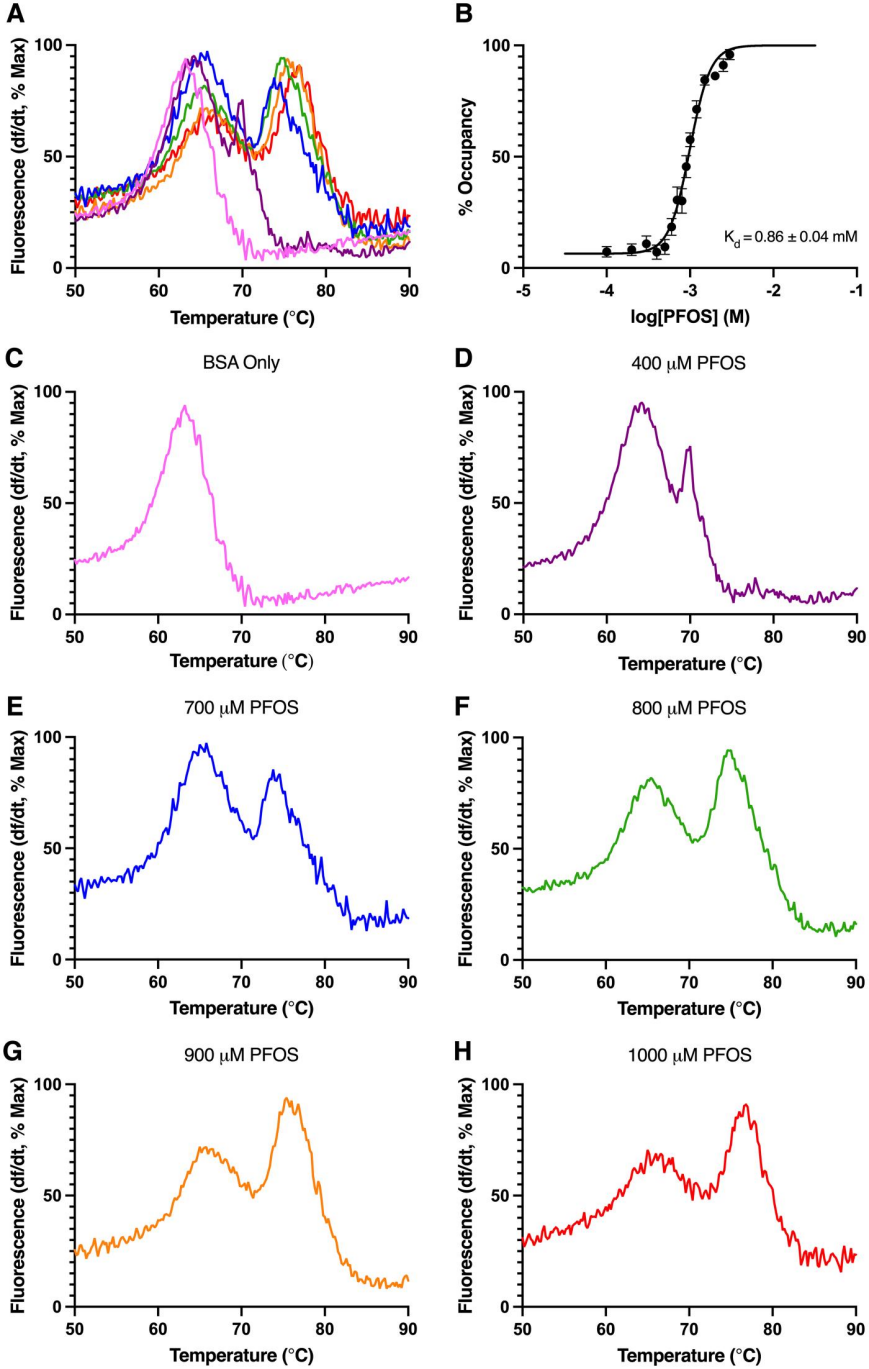
BSA binding of PFOS. (A) Change in fluorescence for BSA with increasing concentrations of PFOS. Increasing concentrations of PFOS are depicted by increasing wavelength of color from violet (0.4 mM) to red (1 mM). (B) The percent occupancy of BSA binding to PFOS, as a function of the log-transformed concentration of PFOS. (C–H) Melt curves for BSA at individual concentrations of PFOS from 0 to 1000 μM. N = 9 replicates.

For each albumin, DSF was used to detect differences in *T*_M_ with increasing concentrations of each PFAS congener, and to estimate binding affinity values, reported in Table 2. Maximum Δ*T*_M_ values ranged from 3.53°C to 15.9°C in HSA, 10.5°C to 17.2°C in BSA, 5.74°C to 13.9°C in PSA, and 4.76°C to 12.2°C in RSA (Table 2). Binding affinities of PFAS ranged from 0.41 to 2.64 mM for HSA, 0.80 to 3.06 mM in BSA, 0.37 to 2.12 mM for PSA, and 0.33 to 1.90 mM for RSA (Table 2). Shown in Figure 3 are representative concentration-response analyses determining PFBS binding by HSA (Figs. 3A and 3B), BSA (Figs. 3C and 3D), PSA (Figs. 3E and 3F), and RSA (Figs. 3G and 3H). Each albumin bound PFAS congeners with charged functional groups, as evidenced by concentration dependent PFAS-induced shifts in protein melting temperature (Table 2). Consistent with previous findings, no changes in albumin *T*_M_ were detected for any concentration of 6:2 FTOH for any species (Jackson *et al.*, 2021; Table 2). A unique biphasic melt curve in thermal stability change was exhibited for BSA binding of PFOS (Figs. 4A–H), a concentration response pattern consistent with unique binding interactions between PFOS and the BSA protein which is suggestive of cooperativity of binding at different PFAS binding sites (Giancola *et al.*, 1997; Ross and Shrake, 1988; Shrake and Ross, 1988).

**Table 2.** Comparison of PFAS binding affinities across species

Chemical	Chain Length	HSA			BSA				PSA				RSA			
		Δ*T*_m_ (°C)	*K*_d_ (mM)	*R* ^2^	Δ*T*_m_ (°C)	*K*_d_ (mM)	*R* ^2^	*d*	Δ*T*_m_ (°C)	*K*_d_ (mM)	*R* ^2^	*d*	Δ*T*_m_ (°C)	*K*_d_ (mM)	*R* ^2^	*d*
PFBA	4	6.43 (0.43)	2.64 (0.22)	0.96	10.5 (0.18)	3.06 (0.27)	0.93	ns	6.89 (0.07)	1.28* **(0.03)**	0.89	**1.08**	7.06 (0.09)	1.90 (0.09)	0.92	ns
PFHxA	6	10.5 (0.14)	1.71 (0.22)	0.98	17.1 (0.14)	2.17* **(0.06)**	0.98	**0.88**	13.9 (0.14)	0.95*** **(0.02)**	0.97	**2.16**	12.2 (0.09)	1.33 (0.02)	0.97	ns
PFOA	8	13.0 (0.41)	0.79 (0.09)	0.97	14.4 (0.19)	0.99* **(0.01)**	0.92	**2.12**	9.68 (0.07)	0.37*** **(0.01)**	0.89	**7.12**	8.0 (0.13)	0.40*** **(0.01)**	0.97	**0.89**
PFBS	4	11.7 (0.21)	1.68 (0.09)	0.98	15.3 (0.12)	0.80*** **(0.03)**	0.99	**6.02**	12.2 (0.06)	1.25*** **(0.02)**	0.95	**2.41**	10.1 (0.08)	1.41** **(0.03)**	0.96	**1.01**
PFOS	8	15.9 (0.63)	0.69 (0.02)	0.93	15.3 (0.23)	0.86 (0.05)	0.79	ns	5.74 (0.20)	0.38* **(0.06)**	0.85	**2.98**	4.76 (0.38)	0.42 (0.10)	0.87	ns
HFPO-DA	5	13.7 (0.09)	1.57 (0.03)	0.95	12.7 (0.11)	2.55*** **(0.13)**	0.95	**1.99**	10.0 (0.15)	2.12** **(0.03)**	0.93	**1.23**	7.56 (0.10)	1.64 (0.05)	0.94	ns
6:2 FTOH	6	N/A	N/A	N/A	N/A	N/A	N/A	N/A	N/A	N/A	N/A	N/A	N/A	N/A	N/A	N/A
6:2 FTSA	6	3.53 (0.26)	0.41 (0.04)	0.82	17.2 (0.19)	1.39*** **(0.04)**	0.81	**15.3**	8.72 (0.09)	0.52 (0.03)	0.93	ns	7.99 (0.10)	0.33 (0.02)	0.99	ns

Maximum change in melting temperature values, represented as Δ*T*_m_ (±SEM) (°C), and protein binding affinities for each congener, represented as *K*_d_ (±SEM) (mM). Bolded *K*_d_ values represent values significantly different from HSA in GLM with Sidak’s multiple comparisons test.

* *p* < .05,

** *p* < .01,

*** *p* < .001. For comparisons in which significant differences were observed, Cohen’s d (*d*) effect size values are reported, with bolded values denoting a large effect size. Each compound was run on at least 2 separate plates with *n* ≥ 5.

Abbreviations: N/A, nonbinding; ns, nonsignificant result.

#### Interspecies comparison in binding affinities

Interspecies differences in calculated *K*_d_ values were evaluated with GLM, which revealed an overall effect of PFAS that accounted for 21.3% of model variance (χ^2^=815, *p*<.0001). Differences of species accounted for 5.72% of variance (χ^2^=197, *p*<.0001), and the interaction between PFAS and species accounted for 57.5% of variance (χ^2^=149, *p*<.0001).

Human albumin binding affinity for PFBA was decreased 69.4% compared with PSA (*t* = −3.04, *p*=.016; Table 2), but no differences in binding affinity were detected for BSA (*t* = 1.22, *p*=.55) or RSA (*t* = −1.84, *p*=.21). Compared with HSA, binding affinity of PSA for PFHxA was increased 57.1% (*t* = −4.4, *p*=.00063) and BSA affinity was decreased 23.7% (*t* = 3.08, *p*=.016; Table 2), but there were no observed differences for RSA binding (*t* = −2.36, *p* = .079). Overall effects of protein were also detected for PFOA binding. Compared with HSA, PSA binding affinity for PFOA was increased 72.4% (*t* = −6.82, *p*<.0001), and RSA binding affinity was increased 65.5% (*t* = −5.49, *p*<.0001). By contrast, BSA binding affinity of PFOA was 22.5% lower than HSA (*t* = 3.21, *p*=.011).

Human albumin bound PFBS with 71.0% lower affinity (*t* = −10.6, *p*<.0001) compared with BSA, 29.4% lower affinity compared with PSA (*t* = −5.89, *p*<.0001), and 17.5% lower affinity compared with RSA (*t* = −3.68, *p*=.004; Table 2). Porcine albumin binding affinity of PFOS was increased 57.9% compared with HSA (*t* = −2.72, *p*=.032; Table 2), whereas no differences in PFOS binding affinity were detected for BSA (*t* = 1.41, *p*=.43) or RSA (*t* = −2.38, *p*=.072) compared with HSA.

Binding affinity of HSA for the perfluoroalkyl ether acid (PFEA) congener, HFPO-DA, was 47.6% greater than BSA (*t* = 6.86, *p*<.0001), and 29.8% greater than PSA (*t* = 3.79, *p*=.0028; Table 2), but was not different in RSA (*t*=.45, *p*=.96). The relative affinity of HSA for 6:2 FTSA, a fluorotelomer sulfonic acid, was increased 108.9% compared with BSA (*t* = 14.7, *p*<.0001; Table 2), whereas differences in 6:2 FTSA binding affinity were not detected for PSA (*t* = 2.31, *p*=.09) or RSA (*t* = −1.63, *p*=.31).

Comparing the impacts of chain length for a given subclass, GLM revealed significant differences in all species for albumin binding affinity of 4 and 8 carbon PFCA and PFSA congeners. No differences were detected for BSA binding of PFSAs (*t* = −.37, *p* = 1.0). The affinity of HSA binding of PFOA was 107.9% greater than PFBA (*t* = 7.0, *p*<.0001), and 83.5% greater for PFOS than PFBS (*t* = 4.01, *p* = .034; Table 3). For BSA (*t* = 8.36, *p*<.0001) and PSA (*t* = 4.07, *p*=.028), binding affinity for PFOA was 102.2% and 110.3% greater than PFBA, respectively (Table 3), with a similar 106.7% increase in PSA affinity for PFOS compared with PFBS (*t* = 3.97, *p*=.04). For RSA, affinity for PFOA was 130.4% greater than for PFBA (*t* = 4.69, *p*=.0021), and binding affinity for PFOS was 108.2% greater than PFBS (*t* = 4.50, *p*=.0049; Table 3).

**Table 3.** Comparison of 4- and 8-carbon PFAA-binding affinities within species

Protein	*t* ratio	Congener 1	*n*	Congener 1 *K*_d_ (mM)	Congener 2	*n*	Congener 2 *K*_d_ (mM)	*p*	Cohen’s d
HSA	7.00	PFBA	6	2.64 (0.22)	PFOA	6	0.79 (0.09)	**<.0001**	**3.77**
	4.01	PFBS	6	1.68 (0.09)	PFOS	5	0.62 (0.02)	**.034**	**2.77**
BSA	8.36	PFBA	12	3.06 (0.27)	PFOA	6	0.99 (0.01)	**<.0001**	**3.52**
	−0.37	PFBS	6	0.80 (0.03)	PFOS	9	0.86 (0.05)	1.0	ns
PSA	4.07	PFBA	6	1.28 (0.03)	PFOA	9	0.37 (0.01)	**.028**	**3.92**
	3.97	PFBS	6	1.25 (0.02)	PFOS	9	0.38 (0.06)	**.04**	**3.75**
RSA	4.69	PFBA	6	1.90 (0.09)	PFOA	6	0.40 (0.01)	**.0021**	**4.86**
	4.50	PFBS	6	1.41 (0.03)	PFOS	9	0.42 (0.10)	**.0049**	**3.79**

Protein binding affinity values for each 4 and 8 carbon PFCA and PFSA congener, represented as *K*_d_ (±SEM) (mM). *n* denotes the number of replicate *K*_d_ values calculated for each protein/PFAS combination. Bolded *p*-values denote significantly different means between congeners 1 and 2 in GLM with Sidak’s multiple comparisons test. For comparisons in which significant differences are observed, Cohen’s d values of effect size are reported, with bolded values indicating a large effect size.

Abbreviation: ns, nonsignificant result.

#### Interspecies amino acid sequence alignment

SeqAPASS level 1 analysis, which compares primary sequence alignment, showed that the complete primary amino acid sequence of HSA was 79.5% similar to BSA, 78.5% similar to RSA, and 77.8% similar to PSA. Consistent with experimental determination of binding affinity by DSF, SeqAPASS level 1 analysis predicted that each albumin has similar ligand-binding interactions compared with HSA (Table 4, Supplementary Figure 1).

**Table 4.** SeqAPASS level 1 and 3 analysis results

Albumin species	% Similarity overall	Sudlow site I susceptibility match	Sudlow site II susceptibility match	Trp214 susceptibility match
BSA	79.5%	No	Yes	Yes
RSA	78.5%	Yes	Yes	Yes
PSA	77.8%	Yes	Yes	Yes

Percentage of overall amino acid sequence similarity to HSA and evaluation of comparative susceptibility for chemical interactions at key binding sites. ‘Yes’ denotes binding interactions predicted to be similar to HSA, ‘No’ denotes binding interactions predicted to be dissimilar to HSA.

Key amino acid residues involved in polar and electrostatic interactions between HSA and PFAS were further evaluated using level 3 SeqAPASS analysis, which compares individually selected amino acid residues. Level 3 analysis was constrained to include residues in 2 well-characterized binding sites, Sudlow sites I and II, which are involved in HSA binding of drugs, fatty acids, and some PFAS (Ascenzi and Fasano, 2010; Chen and Guo, 2009; Sudlow *et al.*, 1975, 1976). The amino acid residues of the Sudlow II sites of HSA, PSA, BSA, and RSA were identical, demonstrating strong structure and functional conservation across species (Table 4 and Supplementary Tables S2 and S3). Sudlow site I was more divergent relative to HSA; however, PSA and RSA were predicted to have chemical binding capabilities similar to HSA due to the functional conservation of amino acid substitutions (Supplementary Table S2). In contrast, Sudlow site I of BSA, which contains 4 functionally conserved amino acid substitutions and a F211L substitution expected to alter functionality of the protein, was predicted to have different binding capabilities than HSA. Computational substitution of the phenylalanine residue in the Sudlow I site of HSA with a leucine residue (F211L), representing a key amino acid substitution found in BSA, was predicted to be destabilizing to HSA with a −1.97 kcal/mol enthalpy change (ΔΔ*G*).

#### Molecular docking predictions

**Figure 5.**
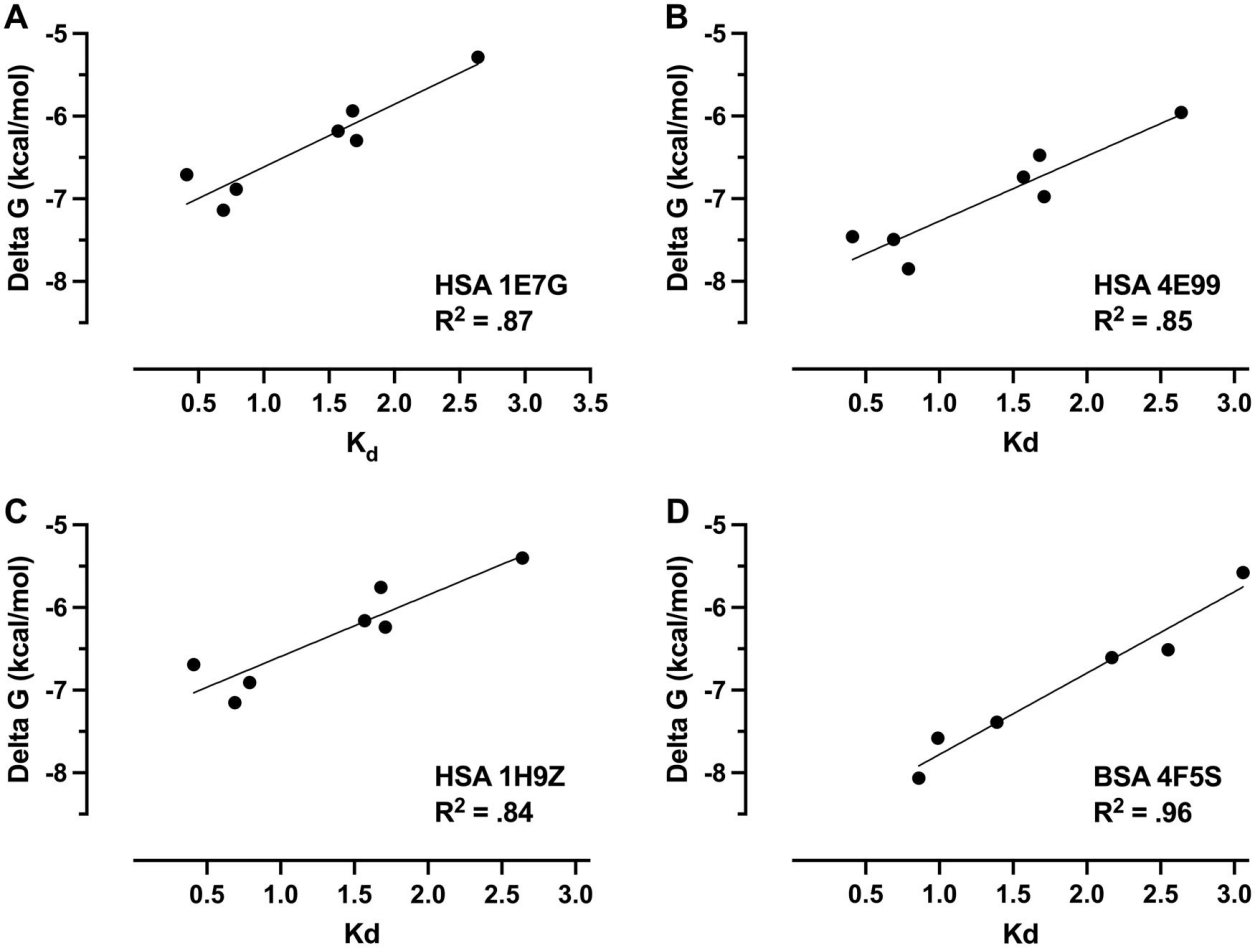
Correlations between DSF-measured dissociation constants (*K*_d_) and computational predictions of Δ*G* of binding different PFAS congeners. (A) HSA 1E7G (conformation bound to myristic acid), (B) HSA 4E99 (conformation bound to PFOS), (C) HSA 1H9Z (intermediate conformation), and (D) BSA 4F5S (native protein with no ligand) without PFBS.

To assess concordance of experimentally derived albumin-PFAS binding affinity analysis and computationally predicted values, Autodock Vina was used to predict enthalpy changes in the free energy of binding (Δ*G*, kcal/mol) for 3 different HSA structural conformations and 1 BSA structure for 7 PFAS congeners analyzed (Supplementary Table S4). Pearson’s correlation was calculated to assess the linear relationship between experimental *K*_d_ values and molecular docking Δ*G* predictions for each PFAS-protein combination (Figure 5). Across HSA structures, strong positive correlations between variables were found for conformations 1E7G (Figure 5A; r(12) = .93, *p* = .002), 4E99 (Figure 5B; r(12) = .92, *p*=.003), and 1H9Z (Figure 5C; r(12)=.91, *p*=.004). With the exception of PFBS, there was also a strong correlation between the variables for all tested congeners in the BSA conformer 4F5S (Figure 5D; r(10)=.98, *p*=.0006).

**Figure 6.**
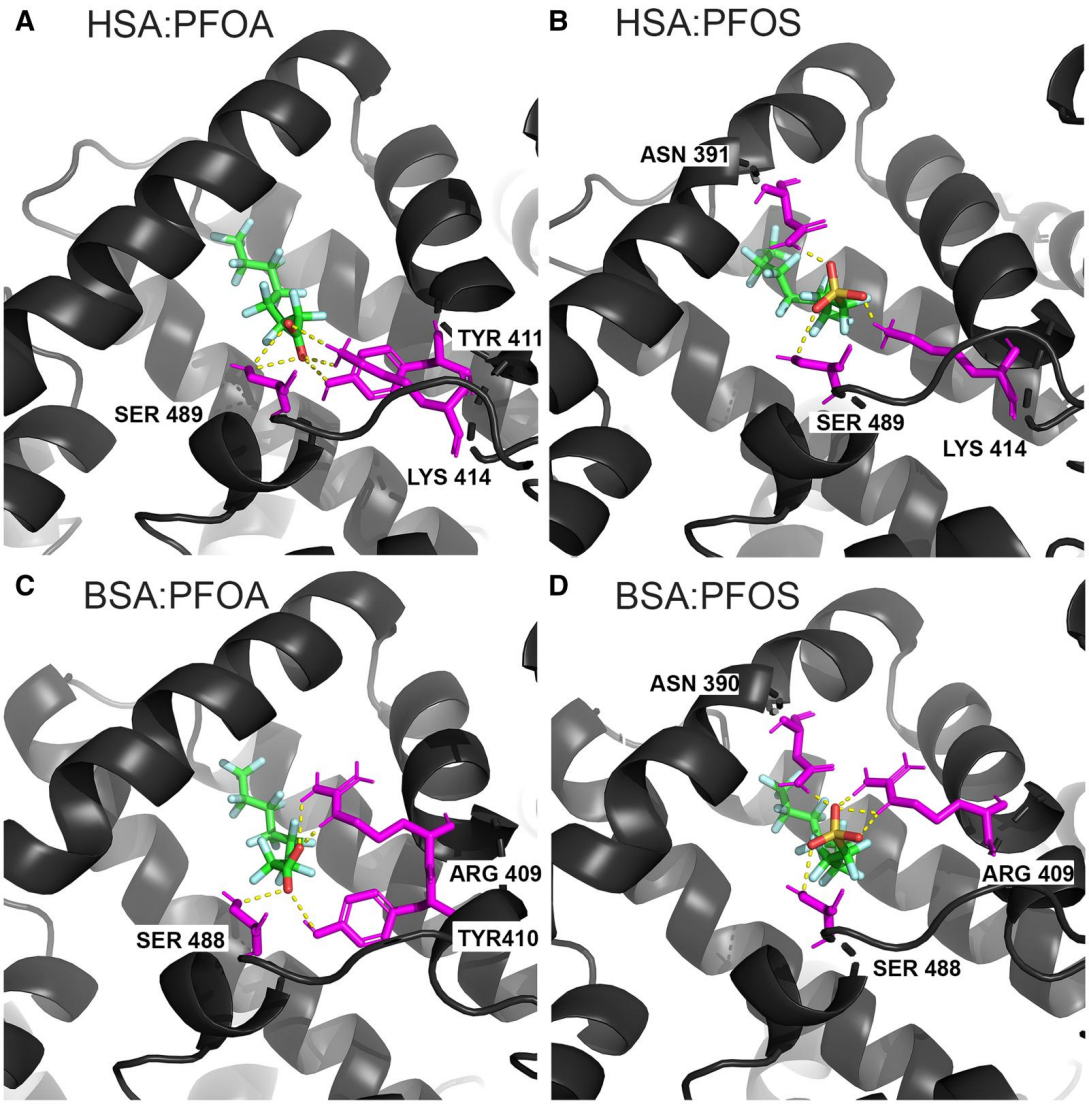
Predicted lowest energy binding conformations (model pose 1) for serum albumin binding to PFOS and PFOA in fatty acid sites 3 and 4 (Sudlow II site) of serum albumin subdomain IIIA. The docked positions of (A) HSA (PDB 4e99) binding to PFOA (Δ*G* = −9.6) and (B) PFOS (Δ*G* = −8.8) and (C) BSA (PDB 4f5s) binding to PFOA (Δ*G* = −9.5) and (D) PFOS (Δ*G* = −9.8) are shown. Serum albumins are shown in black and amino acid residues forming hydrogen bonds with the ligand in each docked conformation are shown in magenta and labeled. Yellow dash marks represent hydrogen bonds between amino acid residues and PFAS ligands. Bound PFAS ligands are colored by atom, with carbons shown in green, fluorines shown in light blue, oxygens shown in red, and sulfurs shown in dark yellow. Three-dimensional structural images were generated with PyMOL.

Although Δ*G* of binding predictions were similar across protein conformations for each PFAS in our analysis, differences were observed in the amino acid residues predicted to be involved in polar interactions with PFAS ligands for the top 9 binding modes at each site analyzed. Molecular docking results demonstrating differences in ligand positioning of PFOS and PFOA when bound in their lowest energy binding conformations by HSA and BSA at fatty acid sites 3/4 (Sudlow site II) are shown in Figure 6, and at fatty acid site 5 shown in Supplementary Figure S2. Binding interactions at Sudlow site II demonstrate that while Δ*G* of binding predictions are similar across proteins, and many of the key binding residues identified in this site are conserved across proteins, subtle changes in protein conformation during ligand binding can lead to compensatory changes in the mode of binding at each site (Figure 6; Supplementary Table S5).

## Discussion

A novel DSF assay was used in this study to compare interspecies differences in albumin binding for an environmentally relevant set of PFAS congeners, to fill critical data gaps and decrease uncertainty in animal to human extrapolation. Protein binding affinities for PFAS are an important determinant of bioaccumulative potential, and a lack of understanding of serum albumin interactions with a majority of PFAS congeners is a major data gap in the field (Cheng *et al.*, 2021; Hall and Peng, 2020). Most experimental research used to understand PFAS accumulation and toxicity has been conducted in animal models, often presenting a need for extrapolation of data to predict human outcomes. Important improvements have been made in the accuracy of computational predictions of toxicity-related factors for PFAS, however absence of experimental data for many chemicals remains a challenge to the development of robust *in silico* frameworks that improve translational predictive capability (Cheng and Ng, 2017; Cheng *et al.*, 2021; Chou and Lin, 2019, 2021; Deepika *et al.*, 2021). Due to differences between the physiochemical properties of PFAS and other persistent organic contaminants, the lack of fundamental physiochemical data for PFAS remains a major limitation in model performance, particularly for the 1000s of PFAS congeners lacking experimental data.

Our analyses demonstrated that each PFAS congener that was bound by HSA was also bound by bovine, porcine, and rat albumin. Those results agreed with sequence homology predictions indicating that HSA, BSA, PSA, and RSA should have similar ligand-binding properties. Although data reporting affinities of BSA and RSA for PFAS are limited, the *K*_d_ estimates derived using our high-throughput DSF platform were within the same order of magnitude as published albumin-PFAS binding affinities (Han *et al.*, 2003; Jackson *et al.*, 2021; Klevens and Ellenbogen, 1954; MacManus-Spencer *et al.*, 2010). It is notable that absolute binding affinities vary greatly across studies due to differences in experimental conditions. As expected, our DSF-determined binding affinities were lower than those reported in studies using other methods; those findings are due to the fact that DSF estimates binding affinities over a range of temperatures, whereas most other approaches estimate binding affinity at a single, typically lower temperature (Jackson *et al.*, 2021; Vivoli *et al.*, 2014). Further, due to differences in experimental conditions across studies, it is not informative to directly compare binding affinity constant values of a given congener that was determined using a different methods (Gao *et al.*, 2020). However, as discussed in our previous work, the use of Δ*T*_m_ in our binding affinity calculations has the advantage of giving a more complete view of the thermodynamic system when comparing binding across compounds (Jackson *et al.*, 2021).

Confirming our earlier findings that fluorotelomer alcohols were not bound by HSA, the 3 additional mammalian serum albumins studied here likewise did not bind 6:2 FTOH. We interpret this lack of observed binding to support the essential influence of a charged functional headgroup as a key structural determinant of albumin-PFAS binding (Jackson *et al.*, 2021). Multiple lines of evidence support this conclusion, including *in vivo* studies that found 8:2 FTOH did not accumulate in pig or rat tissues after oral exposure (Fasano *et al.*, 2006; Xie *et al.*, 2020).

Protein binding is an important mediator of PFAS transport to blood-rich tissues, where equilibrium gradients can drive tissue uptake and resulting toxicity (Cheng and Ng, 2017; Ng and Hungerbühler, 2014; Ng and Hungerbuhler, 2015). Fluorotelomer alcohol congeners and other PFAS that are poorly bound by serum proteins are expected to have important differences in toxicokinetic behaviors, including more rapid metabolism to breakdown products, shorter biological half-lives, differences in accumulation in body compartments, and changes in observed elimination rate. Such impacts are demonstrated by the absence of 8:2 FTOH accumulation in pig and rat tissues (Fasano *et al.*, 2006; Xie *et al.*, 2020). Further, Xie *et al.* (2020) found that although 8:2 FTOH did not bioaccumulate, metabolic breakdown products of 8:2 FTOH with charged carboxylic headgroups that are bound by albumin accumulated in tissues of 8:2 FTOH exposed pigs. Our results demonstrating that HSA, BSA, PSA, and RSA bind to 6:2 FTSA but not 6:2 FTOH, along with *in vivo* evidence that 8:2 FTOH does not accumulate in rat and pig tissues, provides strong evidence that PFAS containing a charged functional group have increased bioaccumulative potential in mammalian species compared with their uncharged analogues (Fasano *et al.*, 2006; Xie *et al.*, 2020).

In addition to functional groups, perfluorinated carbon chain length is a key physiochemical property that determines relative HSA binding affinity for PFAS (Bischel *et al.*, 2010; Crisalli *et al.*, 2023; Jackson *et al.*, 2021; Liu *et al.*, 2017; Ng and Hungerbuehler, 2015). Numerous studies have found that HSA affinities for linear PFAAs of increasing chain length display a U-shaped trend with optimal binding between 6 and 9 aliphatic carbons, whereas congeners with fewer (4–5) or more (10–12) aliphatic carbons are bound less tightly (Jackson *et al.*, 2021; Ng and Hungerbühler, 2014; Salvalaglio *et al.*, 2010). To investigate the hypothesis that BSA, PSA, and RSA would also bind optimally to PFAAs with 6–9 aliphatic carbons, we compared relative affinities for PFCA and PFSA congeners with 4 and 8 aliphatic carbons. Binding affinity was increased for PFOA compared with PFBA for all tested albumins, and PFOS was bound with greater affinity than PFBS for all species except BSA. Those results reveal interspecies conservation of the relationship between carbon chain length and albumin binding affinity, and highlight perfluorinated carbon chain length as a critical physiochemical determinant of albumin binding.

Comparative interspecies albumin-PFAS binding data reported in this study offer insight into the suitability of uncertainty factors (UFs) in the estimation of human health exposure limits. In risk assessment, UFs are used to account for the uncertainty of interspecies variability when extrapolating findings from experimental animal research for human relevance, and require an understanding of PFAS toxicokinetic differences across species to best protect human health (Dankovic *et al.*, 2015). However, in the absence of species or chemical-specific data, an UF of 10 is typically used to extrapolate from animal to human data (Dankovic *et al.*, 2015; Dourson *et al.*, 1996). Dissimilar to all other congeners tested, 6:2 FTOH was not bound by albumin of any species tested, demonstrating that the use of a single UF for all PFAS is not appropriate and that chemical or class-specific and data-derived values may be necessary for some pharmacokinetic parameters. Other experimental methods, such as rapid equilibrium dialysis, should be leveraged to evaluate the proportion of protein-bound to unbound PFAS for each congener, accounting for serum albumin binding as well as other plasma binding proteins, in order to more fully understand the influence of protein binding in PFAS toxicokinetics.

**Figure 7.**
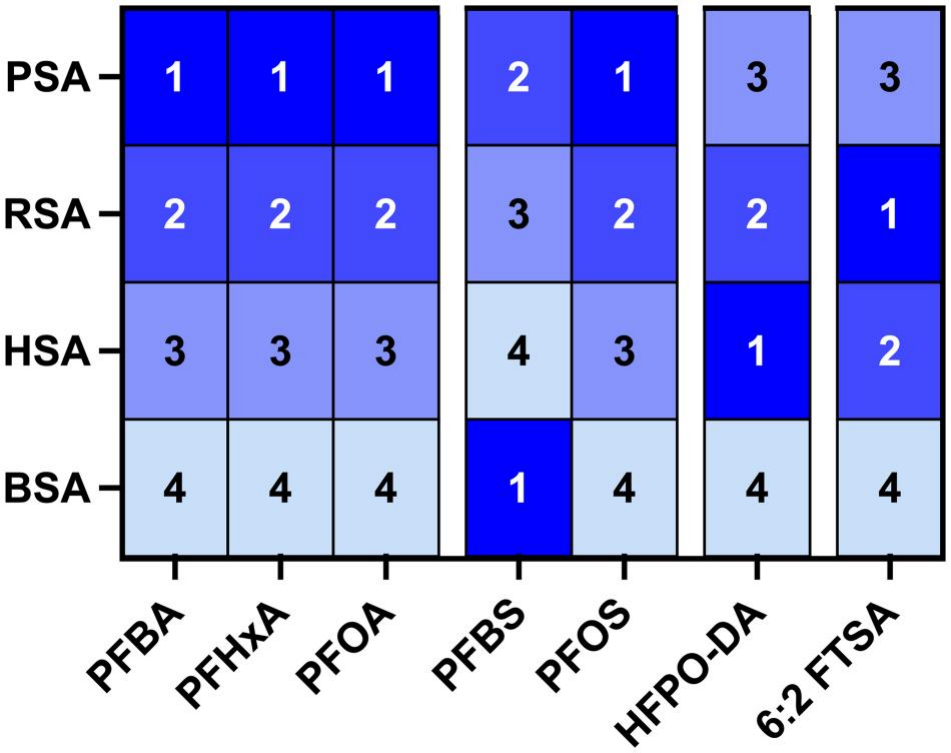
Heatmap showing rank order of binding affinities for each bound PFAS congener. For each congener, 1 indicates highest relative affinity and 4 the lowest affinity for each species.

Beyond the lack of FTOH binding, more subtle differences in species-specific binding affinity emerged. Comparing albumin binding patterns for PFAAs to HSA, our data revealed that PSA and RSA bound this PFAS subclass with increased affinities, whereas BSA bound with decreased affinities, with the notable exception of PFBS (Figure 7). In contrast, relative binding affinity for the perfluoroalkyl ether acid (HFPO-DA) was greatest for HSA, with decreased affinities observed for PSA and BSA binding (Figure 7). Differences in protein flexibility likely explain some binding affinity differences, as albumin undergoes conformational changes to accommodate ligand binding. For example, decreased affinities seen in BSA may be partly explained by the increased rigidity of BSA compared with HSA, which confers less flexibility to accommodate binding (Ketrat *et al.*, 2020).

A notable difference in thermal melt profiles across species was found in BSA-PFOS binding where a biphasic denaturation profile was observed, a profile not seen for other albumin proteins or for BSA with other analyzed PFAS congeners. BSA melted at 2 different temperatures in the presence of PFOS, demonstrating saturation of binding sites (Vivoli *et al.*, 2014). With increasing PFOS concentrations, the proportion of BSA melting at the baseline ligand-free melting temperature decreased, and the remaining proportion of BSA melting at an elevated temperature increased. Biphasic denaturation profiles were also observed in differential scanning calorimetry experiments measuring BSA binding of surfactants (sodium dodecyl sulfate and a dirhamnolipid biosurfactant) (Deep and Ahluwalia, 2001; Giancola *et al.*, 1997; Sánchez *et al.*, 2008), and for HSA binding of palmitic acid (Brandt and Andersson, 1976; Nemergut *et al.*, 2023; Shrake and Ross, 1988). Although several hypotheses have been proposed, there is no consensus as to the cause of biphasic melt curves across protein-ligand affinity studies (Giancola *et al.*, 1997; Luan *et al.*, 2014; Nemergut *et al.*, 2023; Shrake and Ross, 1988). Due to the heterogeneity of binding sites across the 3 homologous domains of the albumins studied, with each site comprising different chemical properties that are optimally suited for different ligands, we hypothesize that PFOS is preferentially binding and stabilizing 1 or 2 of the homologous domains of BSA (Fasano *et al.*, 2005; Nemergut *et al.*, 2023). Another plausible explanation for the cause of the biphasic melt curve is cooperativity, in which binding of BSA to PFOS at 1 ligand binding site changes the conformation of the protein in a way that alters its affinity for PFOS at another binding site (Giancola *et al.*, 1997; Ross and Shrake, 1988; Shrake and Ross, 1988). The hill coefficient of the biphasic melt curve for BSA binding to PFOS (hill slope = 3.5 ± 0.3) offers further evidence of cooperativity, as a hill coefficient greater than 1 is suggestive of cooperative binding (Weiss, 1997).

Observed binding affinity differences are also likely dictated by changes in the amino acid sequence of each albumin, particularly in binding sites. However, details regarding site-specific albumin binding affinity for different PFAS congeners are poorly understood. Albumin binds to several PFAA congeners through non-covalent hydrophobic and electrostatic interactions (Chen *et al.*, 2020; Fasano *et al.*, 2005; Liu *et al.*, 2017; Salvalaglio *et al.*, 2010; Zhang *et al.*, 2009). Proposed albumin binding sites for PFAS include Sudlow sites I and II, interactions impacting Trp214, and several fatty acid binding sites (Cao *et al.*, 2022; Chen and Guo, 2009; D’eon *et al.*, 2010; MacManus-Spencer *et al.*, 2010; Maso *et al.*, 2021; Moro *et al.*, 2022; Salvalaglio *et al.*, 2010; Sheng *et al.*, 2020). Analysis of binding affinity data based on structural changes of HSA Trp214 residue via quenching of intrinsic tryptophan fluorescence, and Sudlow sites I and II via fluorescence displacement of site-specific probes, found that carbon chain length and functional group of PFAA congeners strongly influenced HSA binding capability at each site (Chen and Guo, 2009). Although our DSF assay does not have site-specific experimental resolution, our differential binding, homology, and docking results support the fact that subtle changes in amino acid sequence in putative PFAS binding sites may impact albumin binding affinities for PFAS.

Molecular docking was utilized to further investigate HSA and BSA binding of PFAS, and to compare the dissociation constants determined from our *in vitro* assay results with *in silico* binding predictions from Autodock Vina, which was used to simulate albumin-PFAS binding at 26.85°C. Numerous different amino acid residues have been identified to contribute to serum albumin binding of PFAS (Chen *et al.*, 2015; Chi *et al.*, 2018; Delva-Wiley *et al.*, 2021; Gallagher *et al.*, 2023; Luo *et al.*, 2012; Peng *et al.*, 2024; Salvalaglio *et al.*, 2010; Sheng *et al.*, 2020; Wang *et al.*, 2016; Wu *et al.*, 2024). Across HSA and BSA structural conformations, differences in key amino acid residues involved in hydrogen bonding interactions with each PFAS ligand were observed. For example, Lys414 in Sudlow site II of HSA (PDB 4E99) was predicted to form hydrogen bonds with PFOA and PFOS in 6 of the top 9 binding modes for each ligand, whereas in BSA (PDB 4F5S) this residue was not identified to form hydrogen bonds with either ligand in any of the top 9 binding modes. Previous molecular docking studies support these findings, as 2 other studies have identified Lys414 to be involved in HSA binding to PFOS and PFOA, in both bound (PDB 4E99) (Luo *et al.*, 2012) and unbound (PDB 4K2C) (Chi *et al.*, 2018) conformations. Similarly, 2 previous docking studies simulating PFOS binding at Sudlow site II of 2 ligand-free BSA conformations (PDB 3V03 and Swiss Model BSA P02769) did not identify Lys413 as a key binding residue at this site (Chen *et al.*, 2015; Wang *et al.*, 2016). These findings highlight that although binding affinities for PFAS across albumin proteins are similar, many different modes of binding may contribute to stabilization of PFAS congeners in albumin binding sites.

Like results reported by Ng and Hungerbuhler (2015), predicted binding affinities were increased compared with those determined experimentally. The use of multiple HSA structures allowed binding affinity assessment across different conformations, addressing the limitation that Autodock Vina does not account for changes in protein conformation during ligand binding (Ng and Hungerbuehler, 2015; Trott and Olson, 2009). Across HSA structural conformations, there was a strong positive correlation between experimentally determined dissociation constants and docking predictions. Except for PFBS, there was also a strong positive correlation between these values for BSA binding. Strong agreement of experimental and predicted values provides 2 lines of evidence to support our experimental conclusions and strengthens the hypothesis that subtle interspecies differences in PFAS interactions with amino acids in specific binding sites will influence PFAS binding affinities.

There was poor concordance between the molecular docking prediction for BSA-PFBS binding compared with our experimentally determined affinity, with molecular docking predicting a much lower binding affinity. Tight binding of BSA to PFBS, comparable to binding affinity for PFOS, was also reported in a study utilizing equilibrium dialysis to determine protein-water partition coefficients (Bischel *et al.*, 2011). As seen in our experiments, Bischel *et al.* (2011) also found significantly increased BSA binding affinities for PFCAs with increased aliphatic chain lengths, but not PFSAs. In contrast, 2 other studies on BSA-PFAS binding reported decreased affinity for PFBS compared with PFOS (Alesio *et al.*, 2022; Allendorf *et al.*, 2019). However, Bischel *et al.* (2011) states that some common fluorescence methods may not be suitable for assessing albumin binding to short chain PFAAs, including the fluorescence quenching approach used by Alesio *et al.* (2022), due to the failure of these short-chain PFAAs to cause the conformational changes in secondary structure of BSA that elicit spectral changes (Hebert and MacManus-Spencer, 2010; Qin *et al.*, 2010). Inconsistency between our *in vitro* and *in silico* results for BSA-PFBS binding, as well as the biphasic denaturation profile seen only with BSA-PFOS binding, suggests that PFAS binding by BSA may involve major differences in binding modalities for sulfonic acids. These and other HSA-PFAS binding studies reporting different trends in binding affinities across different structural classes of PFAS (Jackson *et al.*, 2021), or failure of proposed models of HSA-PFAA binding to generate accurate results for short-chain compounds (Hebert and MacManus-Spencer, 2010), also concluded that differences in the mode of binding for these compounds are a likely explanation for divergence from predicted relative binding affinities.

At Sudlow site I of BSA, a single amino acid residue substitution was identified that was predicted by SeqAPASS to alter functionality of this key PFAS-binding site relative to HSA. Sudlow site I is found in albumin’s subdomain IIA, overlaps with fatty acid site 7, and serves as the warfarin binding site, in addition to binding other bulky, heterocyclic anionic compounds (Ascenzi and Fasano, 2010; Fasano *et al.*, 2005). In the mature HSA protein, key residues involved in ligand binding evaluated at Sudlow site I included Tyr150, Glu153, Lys195, Gln196, Lys199, Phe211, Trp214, Ala215, Arg218, Arg222, Leu238, His242, Arg257, His288, and Ala291 (Ascenzi and Fasano, 2010). All of these key residues are functionally conserved in PSA and RSA with respect to HSA. However, the phenylalanine residue (Phe211) found in HSA is replaced with a leucine residue (Leu210) in BSA. Phenylalanine and leucine are both nonpolar and hydrophobic, however phenylalanine is a bulkier, aromatic residue and is likely to introduce more steric hindrance at the binding site than leucine. Calculations using the DUET web-tool supported the SeqAPASS prediction of altered functionality at Sudlow site I of BSA, predicting that a F211L mutation in HSA would yield a destabilizing enthalpy change which would likely alter protein-ligand interactions at this site. Our findings suggest that this destabilizing substitution at Sudlow site I in BSA could alter binding for ligands known to bind this site, including PFBA, PFBS, PFOA, and PFOS (Chen and Guo, 2009).

Binding affinities of PSA for PFCAs and PFSAs were significantly greater than HSA affinities, whereas affinity for HFPO-DA was significantly decreased. Relative to HSA, PSA contains the greatest percentage of amino acid sequence divergence (77.8% homology). This degree of sequence divergence suggests that PSA should exhibit more functional differences compared with HSA than BSA or RSA, which agrees with our experimental findings; PSA binding affinities showed differences from HSA affinities with large effect sizes for all of the PFAA and PFEA congeners (Table 2).

To more fully understand interspecies differences in PFAS distribution and retention, it is necessary to consider other parameters in conjunction with albumin binding affinity, including binding affinities of other proteins, tissue transfer efficiencies, and metabolism (Cheng and Ng, 2017; Chou and Lin, 2019; Gao *et al.*, 2019; LaLone *et al.*, 2013). For many protein-bound ligands, including pharmaceuticals, increased protein binding affinity increases biological half-life (Bech *et al.*, 2018; Sleep *et al.*, 2013; Zorzi *et al.*, 2019). It is more challenging to predict the impacts of albumin binding affinity on perfluorinated compounds because they are not metabolized, unlike most pharmaceuticals. Coupling our data with published findings, we hypothesized that increased binding affinity of PSA for PFAAs will increase PFAA half-life in the blood compartment of pigs. In support of this conclusion, a study conducted by Numata *et al.* (2014) found that blood plasma in pigs has higher affinity for PFAAs, relative to reported affinities for cows and sheep (Kowalczyk *et al.*, 2013; Numata *et al.*, 2014; van Asselt *et al.*, 2013). However, PFBS is the only congener which has a longer elimination half-life in pigs compared with reported values in humans, whereas half-lives for PFOS, PFOA, and PFHxA are longer in humans, highlighting the need to consider more physiochemical parameters in addition to albumin binding (Table 5).

**Table 5.** Reported elimination half-lives of PFAAs in serum/plasma across species and DSF-determined albumin binding affinities

	PFBA		PFHxA		PFOA		PFBS		PFOS	
	Half-life	*K*_d_ (mM)	Half-life	*K*_d_ (mM)	Half-life	*K*_d_ (mM)	Half-life	*K*_d_ (mM)	Half-life	*K*_d_ (mM)
Human	3.1 days^a^	2.64 (0.22)	32 days^b^	1.71 (0.22)	2.3–8.5 years^a^	0.79 (0.09)	25.8 days^a^	1.68 (0.09)	3.3–5.4 years^a^	0.69 (0.02)
Cow	N/A	3.06 (0.27)	N/A	2.17 (0.06)	0.8–1.3 days^c^	0.99 (0.01)	N/A	0.80 (0.03)	39–120 days^c^	0.86 (0.05)
Pig	N/A	1.28 (0.03)	4.1 days^d^	0.95 (0.02)	236 days^d^	0.37 (0.01)	43 days^d^	1.25 (0.02)	1.7 years^d^	0.38 (0.06)
Rat	1–9.2 h^a^	1.90 (0.09)	0.4–9.8 h^e^	1.33 (0.02)	0.1–15 days^a^	0.40 (0.01)	0.6–7.4 h^a^	1.41 (0.03)	24–83 days^a^	0.42 (0.10)

Sources for half-life values are Pizzurro *et al.*, 2019^a^, Lau, 2015^b^, Death *et al.*, 2021^c^, Numata *et al.*, 2014^d^, Russell *et al.*, 2013^e^. Binding affinities represented as *K*_d_ (±SEM) (mM).

Abbreviation: N/A, no reported values.

Understanding differences in PFAS-protein interactions between rats and humans is an important endpoint necessary for accurate translational extrapolation of experimental rodent toxicity studies. The observed differences in PFAS binding affinities between HSA and RSA in this study could serve as important data to decrease uncertainty in the application of animal model data for human relevance, and can be used to help understand differences in PFAS behavior across species. Overall, HSA and RSA were quite similar in binding affinities for most PFAS, and our findings suggest that guideline toxicity studies in rat models are not likely to be greatly impacted by species differences in albumin binding for the PFAS congeners in our test set. PFAS have also been detected widely in the tissues of livestock species, including cows and pigs, and are excreted in dairy milk (Death *et al.*, 2021; Guruge *et al.*, 2008; Numata *et al.*, 2014; Rock *et al.*, 2023; van Asselt *et al.*, 2013). As one of the major routes of exposure to PFAS for humans is consumption of contaminated food and water, assessment of PFAS accumulation in livestock species is also needed to evaluate the role of livestock consumption in human PFAS exposure (Roth *et al.*, 2020; Sunderland *et al.*, 2019). Our findings demonstrate that HSA-PFAS binding is less similar to BSA and PSA, suggesting increased uncertainty in extrapolation of toxicokinetic parameters and distribution of PFAS in cows and pigs. Data generated in our study using the DSF assay provide necessary interspecies comparisons and serve as an essential step toward understanding the physiochemical properties of PFAS across species.

## Supplementary Material

kfae028_Supplementary_Data
