# Supplementary material for: Quantitative cross-species comparison of serum albumin binding of per- and polyfluoroalkyl substances from five structural classes
Source: Toxicol Sci. 2024 Mar 22;199(1):132–49. doi: 10.1093/toxsci/kfae028 (PMC11057469; doi:10.1093/toxsci/kfae028)
Supplement: kfae028_Supplementary_Data [file kfae028_supplementary_data.docx]

**Quantitative Cross-Species Comparison of Serum Albumin Binding of Per- and Polyfluoroalkyl Substances from 5 Structural Classes**

Hannah M. Starnes^1^, Thomas W. Jackson^1,2^, Kylie D. Rock^1,3^, Scott M. Belcher^1*^

1. Department of Biological Sciences

North Carolina State University

127 David Clark Labs Campus Box 7617

Raleigh, North Carolina 27607, USA

1. Current Address:

Public Health and Integrated Toxicology Division

Center for Public Health and Environmental Assessment

U.S. Environmental Protection Agency

Research Triangle Park, NC 27711, USA

1. Current Address:

Department of Biological Sciences

Clemson University

Clemson, SC 29634, USA

**Index:**

1. Table S1: List of abbreviations
2. Table S2: SeqAPASS level 3 analysis for Sudlow Site I
3. Table S3: SeqAPASS level 3 analysis for Sudlow Site II
4. Table S4: Delta G Binding Predictions from Autodock Vina
5. Table S5: Binding comparison at FA sites 3/4 (Sudlow site II)
6. Figure S1: Full protein sequence alignment
7. Figure S2: Molecular docking results at fatty acid site 5

Table S1. List of abbreviations

| **Abbreviation** | **Term** |
| --- | --- |
| 6:2 FTOH | 6:2 fluorotelomer alcohol |
| 6:2 FTSA | 6:2 fluorotelomer sulfonic acid |
| BSA | Bovine serum albumin |
| DSF | Differential scanning fluorimetry |
| HBS | HEPES-buffered saline |
| HFPO-DA or GenX | Hexafluoropropylene oxide-dimer acid |
| HSA | Human serum albumin |
| OA | Octanoic acid |
| PBPK | Physiologically based pharmacokinetic |
| PDB | Protein Data Bank |
| PFAAs | Perfluoroalkyl acids |
| PFBA | Perfluorobutanoic acid |
| PFBS | Perfluorobutanesulfonic acid |
| PFCAs | Perfluorocarboxylic acids |
| PFDA | Perfluorodecanoic acid |
| PFHxA | Perfluorohexanoic acid |
| PFHxS | Perfluorohexanesulfonic acid |
| PFNA | Perflurononanoic acid |
| PFOA | Perfluorooctanoic acid |
| PFOS | Perfluorooctanesulfonic acid |
| PFSAs | Perfluorosulfonic acids |
| PSA | Porcine serum albumin |
| RSA | Rat serum albumin |
| SeqAPASS | Sequence Alignment to Predict Across Species Susceptibility |


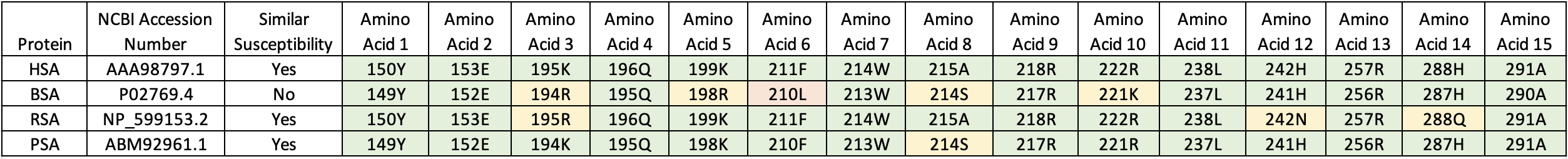
Table S2. SeqAPASS level 3 analysis for Sudlow Site I.

Sequence alignment of amino acids in Sudlow site I, and SeqAPASS classification of susceptibility for ligand binding compared to HSA. Green boxes indicate identical amino acids compared to HSA, yellow boxes indicate partial (functionally conserved) matches to HSA, and red boxes indicate amino acids that do not match HSA.

Table S3. SeqAPASS level 3 analysis for Sudlow Site II.


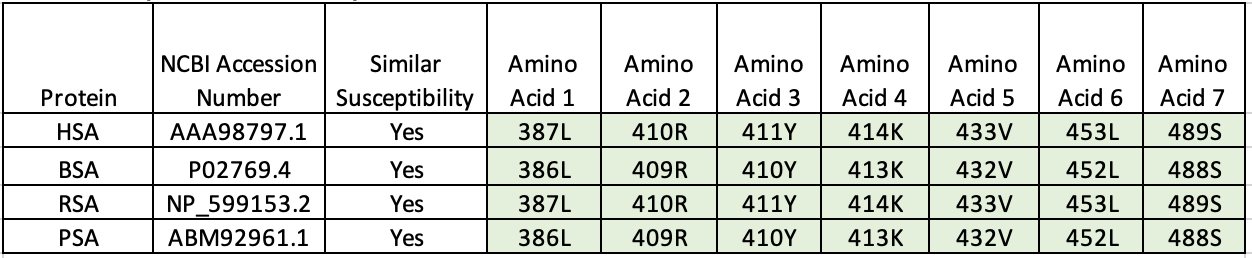


Sequence alignment of amino acids in Sudlow site II, and SeqAPASS classification of susceptibility for ligand binding compared to HSA. Green boxes indicate identical amino acids compared to HSA.

Table S4. Delta G of Binding Predictions from Autodock Vina


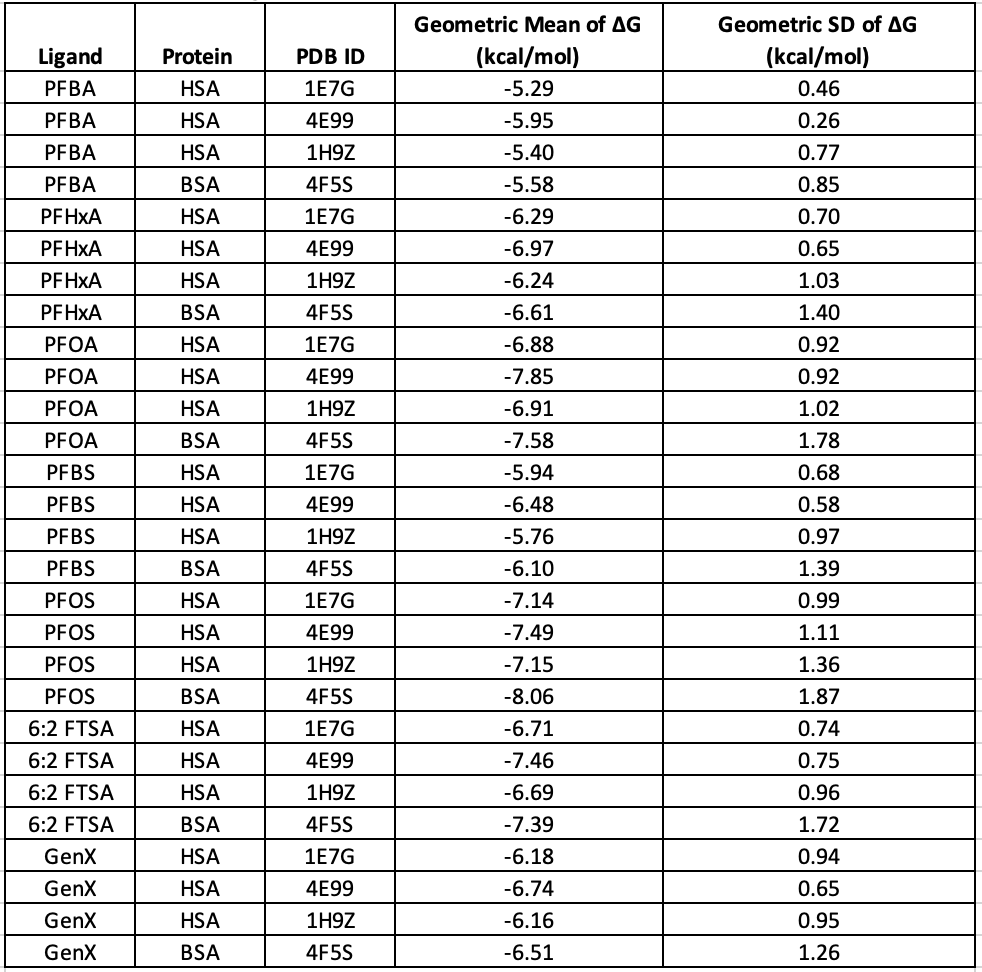


Geometric mean and standard deviation of Autodock Vina predicted ΔG of binding across six binding sites, in three high-resolution structural conformations of HSA and one of BSA.

Table S5. Binding comparison at FA sites 3/4 (Sudlow site II)
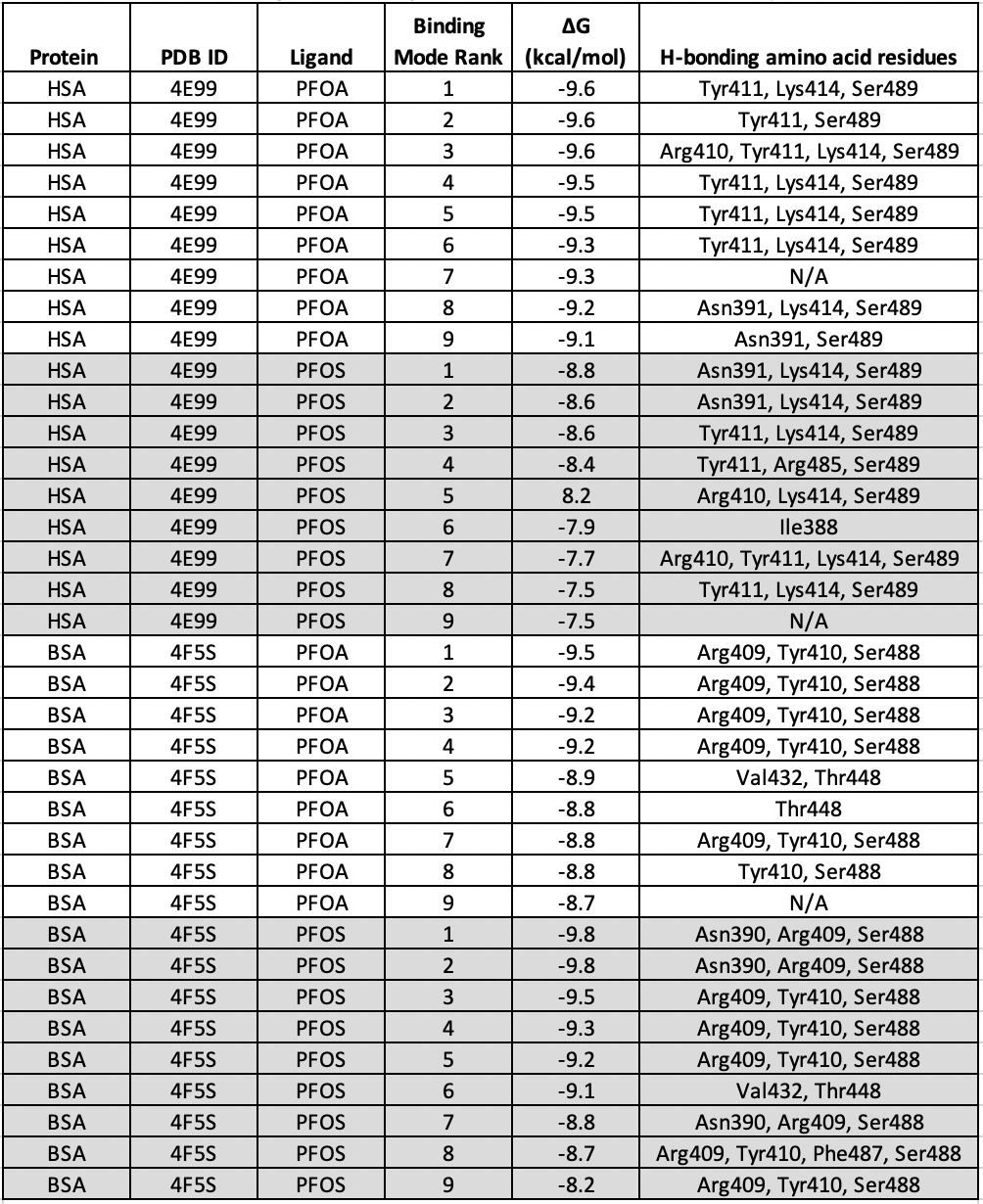


Autodock Vina predictions of HSA (PDB 4E99) and BSA (4F5S) binding to PFOA and PFOS at fatty acid sites 3/4 (Sudlow site II). ΔG of binding and key amino acid residues forming hydrogen bonds with the ligand are shown for each of the top 9 predicted binding conformations.


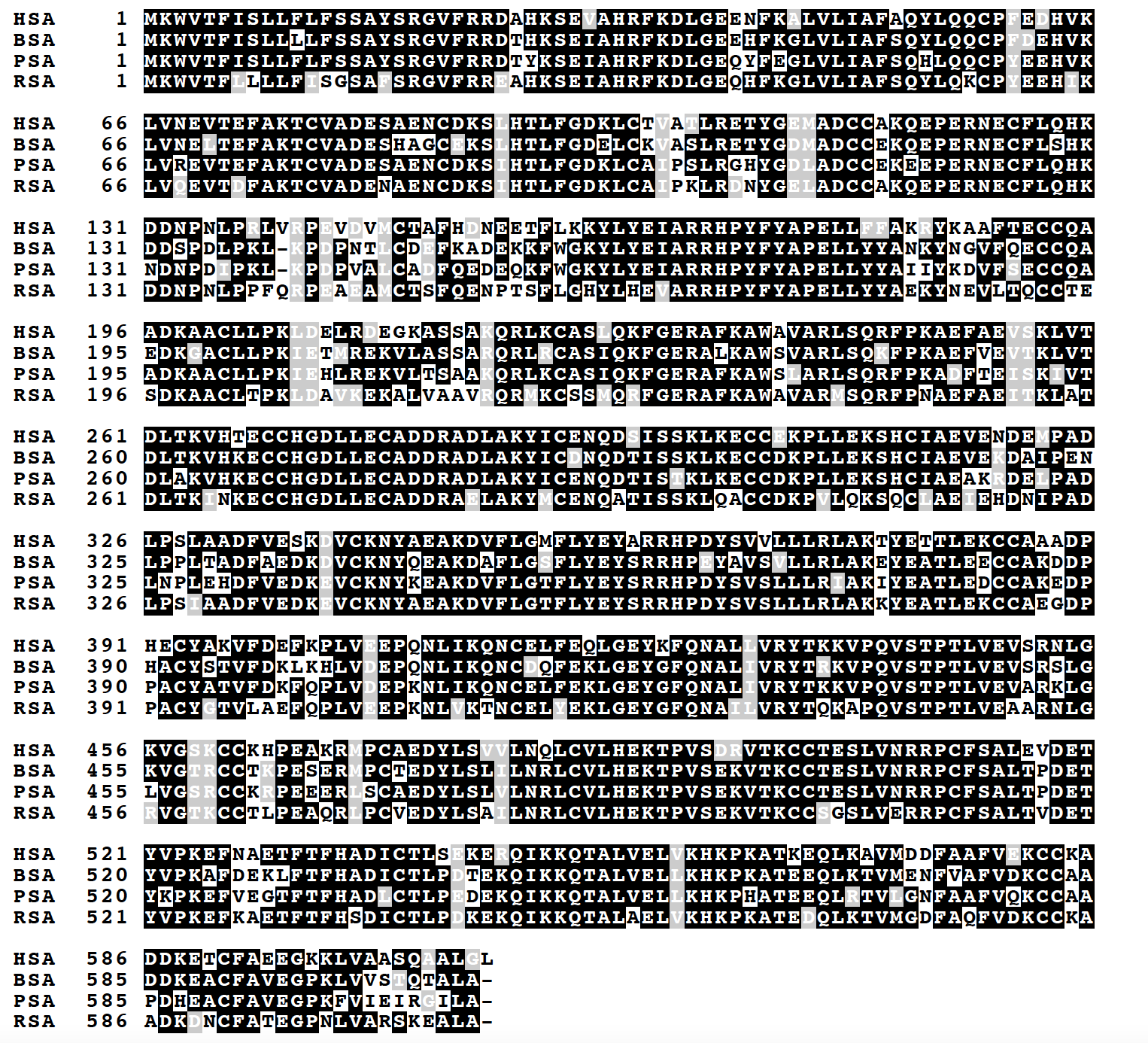


Figure S1. Full protein sequence alignment of the 609 amino acids in serum albumin, including the 24 amino acids in the prepro protein and the 585 amino acids in the mature albumin protein. Black background indicates identical residues, gray background indicates similar residues, and white background is used for remaining residues. Sequence alignment generated by T-Coffee (<https://tcoffee.crg.eu/apps/tcoffee/do:regular>).


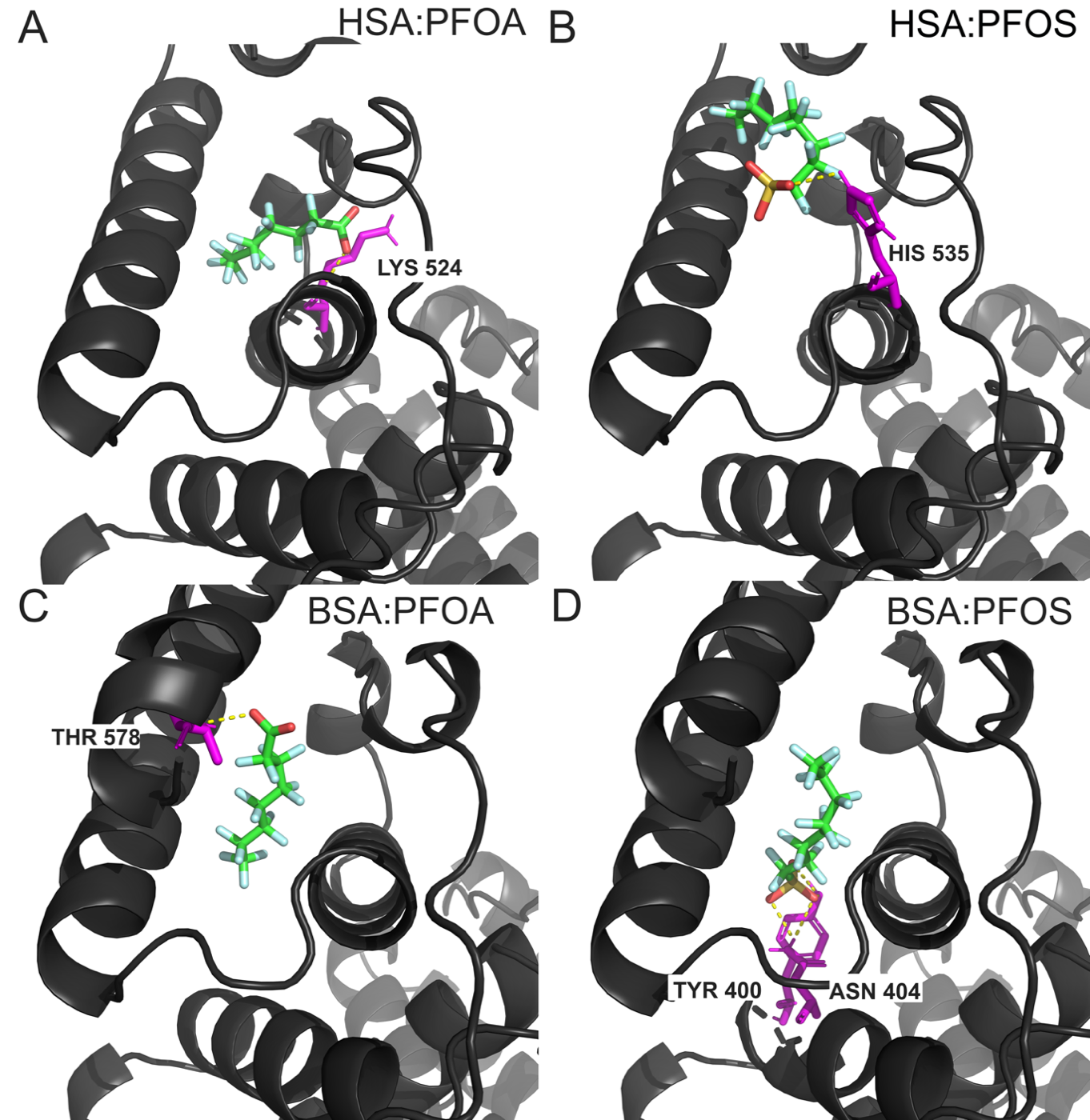


Figure S2. Predicted lowest energy binding conformations (model pose 1) for serum albumin binding to PFOS and PFOA in fatty acid site 5 of serum albumin subdomain. IIIB. The docked positions of A) HSA (PDB 4e99) binding to PFOA (ΔG = -7.2) and B) PFOS (ΔG = -6.7) and C) BSA (PDB 4f5s) binding to PFOA (ΔG = -9.7) and D) PFOS (ΔG = -10.2) are shown. Serum albumins are shown in black and amino acid residues forming hydrogen bonds with the ligand in each docked conformation are shown in magenta and labeled. Yellow dash marks represent hydrogen bonds between amino acid residues and PFAS ligands. Bound PFAS ligands are colored by atom, with carbons shown in green, fluorines shown in light blue, oxygens shown in red, and sulfurs shown in dark yellow. Three-dimensional structural images were generated with PyMOL.
